# The Effect of Composition, Pre-Treatment on the Mechanical and Acoustic Properties of Apple Gels and Freeze-Dried Materials

**DOI:** 10.3390/gels8020110

**Published:** 2022-02-10

**Authors:** Ewa Jakubczyk, Anna Kamińska-Dwórznicka, Ewa Ostrowska-Ligęza

**Affiliations:** 1 Department of Food Engineering and Process Management, Institute of Food Sciences, Warsaw University of Life Sciences, 02-776 Warsaw, Poland; anna_kaminska1@sggw.edu.pl; 2 Department of Chemistry, Institute of Food Sciences, Warsaw University of Life Sciences, 02-787 Warsaw, Poland; ewa_ostrowska_ligeza@sggw.edu.pl

**Keywords:** agar gel, texture, mechanical properties, acoustic properties, porosity, glass transition temperature

## Abstract

This study aimed to determine the effect of the addition of apple juice concentrate (AJC) on the properties of agar gel and dried materials. Agar gels with the addition of apple juice concentrate in the range of 5–20% were prepared with or without the addition of maltodextrin. The gels were also soaked in the solution of AJC. The water content, water activity, densities, some mechanical and acoustic descriptors of gels, and the freeze-dried gels were analysed. The porosity and shrinkage of dried products were also investigated. The addition of AJC significantly changed mechanical and acoustic properties of gels. The hardness of gels decreased with a higher addition of concentrate. Dried samples with a lower concentration of sugars (the lower addition of AJC) were characterised by lower shrinkage and higher porosity, as well as crispness and glass transition temperature. The investigated mechanical and acoustic properties of dried gels showed the addition of apple concentrate at the level of 5% to agar solution was optimal.

## 1. Introduction

Gels play an important role in the production of many food materials such as jelly or dairy products [1]. The addition of sugars and other dry ingredients to gels may significantly modify their taste and storage stability due to a decrease in water content and water activity [2]. Gelled products can be also created with addition of fruit purees and juices. The properties of gel with the addition of mango pulp as sweetening and texturizing agents were analysed [3]. Some examples when the fruit or vegetable juices were added to hydrocolloid solutions to prepare novel gelled products can be found in the literature [3,4,5,6]. However, the fabrication of dried snacks based on fruit concentrates and purees has been less common [7,8,9], but some studies indicated that the application of drying fruit materials enabled to obtain healthy products with attractive appearances and textures [7,8,10].

Drying is a popular unit operation frequently applied to reduce water content to a level which enables the storage of food for a long time. This technology prevents microbial contamination and slows down the enzymatic and non-enzymatic reactions which can take place in food [11,12]. Many fruit snacks have been produced with application of a freeze-drying technique. Drying at low a temperature and pressure requires a longer time of process than other drying methods [11]. However, the quality of products obtained by sublimation of frozen material is very high. The shape and structure of freeze-dried products can be protected. Additionally, the method creates products with a high retention of nutrients and flavours [13]. There are some studies that describe the properties of dried materials produced with different gelling hydrocolloids.

The common hydrocolloid used in the creation of food gels is agar-agar which can form a thermo-reversible and stable gel over a wide range of temperature [4,14]. Additionally, compared with other hydrocolloids such as gelatine, agar is suitable for vegetarians because of its non-animal source of origin [15].

The application of drying to hydrocolloids gels results in the formation of a cellular solid with a characteristic porous structure which can be a carrier for food ingredients such as minerals, vitamins, and flavouring components [16]. The production of dried gels with addition of other ingredients can be a method to obtain a wide range of food snack or fruit bars [8]. Some studies have shown that the application of different drying methods as well as the aeration of an agar solution with apple puree modified the sorption properties and structure as well as stability of dried agar gels during storage [9]. The incorporation of fruit ingredients (concentrates, pulps, and juices) to a gel structure can be a tool to obtain products with a tailored texture, density and colour [4,17,18].

The texture of gels can be characterised by many different mechanical and acoustic attributes perceived by consumers [19]. The textural properties of gelled products can be analysed using different instrumental techniques. The large strain deformation method is recommended to obtain fracture descriptors because they are strongly related to sensory attributes of texture [20]. The texture of food can be also predicted based on the acoustic emission which can be generated during deformation. Acoustic emission methods have been applied to investigate the properties of many crispy foods such as extrudates [21,22], dried gels [23], and sugar foams [24,25]. The structure of the material and composition of foods may influence their acoustic properties. The fractures generated during the deformation of a material can be a source of an acoustic signal which may contain information about the texture of products [26]. The combination of mechanical and acoustic properties can provide more information about the texture of food than any instrumental method alone [27].

The addition of fruit juices or concentrates as liquid ingredients instead of fruit puree (suspension of semi-liquid plant material with solid particles) to a gel matrix may lead to a new structure of final product. Another important approach is to try and control the texture of dried gels with fruit products. However, up to this point, little research has been performed in this area [8].

Our previous study showed that the addition of chokeberry juice concentrate and a foaming agent at different concentrations modified the structure (size of bubbles and their distribution) as well as texture of foamed gels (marshmallows) [17]. Therefore, the effect of the composition of gel with fruit concentrate and the pre-treatment of agar gel (e.g., osmotic dehydration) on the texture of dried materials can be crucial in a new product design and development.

This study aimed to determine the effect of the addition of apple juice concentrate, soaking of agar gel in AJC solution, and the application of freeze drying on the mechanical and acoustic properties of agar gel with or without maltodextrin.

## 2. Results and Discussion

### 2.1. Characteristics of Apple Gels

Pure agar gels (samples prepared only with a hydrocolloid addition as a gelling agent), products obtained by gelling the mixture of agar solution with apple juice concentrate (with or without maltodextrin), as well as agar gel and agar-maltodextrin gels soaked in the osmotic solution of AJC were analysed. The overall appearance of gels after solidification in Petri dishes and 24 h of storage was checked (visual observation). The presence of diluent on the surface of gels indicated that the syneresis occurred. This means that some amount of water was expelled from the gel. The syneresis was not observed for gels (C) with the addition of apple juice concentrate (AJC) in the range of 5–15%, also for samples (C-DE) prepared with AJC and maltodextrin. The increase in amount of AJC to 20% led to an instable gel with released liquid on its surface. Additionally, it was difficult to remove the sample from the dish without destroying the gel structure. For this reason, the properties of gels with the addition of 20% AJC were not analysed. However, the agar gels and samples with the addition of maltodextrin soaked (OD) in the solution of AJC with concentration in the range 5–20% showed negligible syneresis.

The addition of AJC to sol solution in the range of 5–15% (with or without maltodextrin) caused a decrease in water content in gels from 93.6 to 83.4% (Table 1). The decrease in water content in gels was related to the addition of higher amount of concentrate and incorporation of a higher concentration of sugars present in AJC. The gels soaked in AJC solutions also contained a lower amount of water in comparison with agar gel (0%C). The soaking of materials in sugar solution caused the dehydration of gels (Table 1). The concentration of sugars in the solution of AJC was in the range from 5 to 20% which was lower than the typical concentration of osmotic agents applied in the dehydration of fruits (25–60%) [28]. However, the osmosis processes for some vegetables were carried out in a concentrated solution of 10% sugars [29]. The slight reduction in water content due to the diffusion of water form gel to AJC solution was observed for gels with maltodextrin (C-DE), the water content decreased from 93.8 to 85.3% with increasing concentration of sugars in the AJC solution.

The water activity of gels ranged from 0.983 to 0.994 but significant differences were observed only for samples with the highest and the lowest addition of AJC or with the application solution with 5 or 20% of sugars. The slight differences between water activity for most gels can be related to the similar availability of water in all gelled samples. Water content in food does not provide information about the nature of water (bond, inherent, or occluded). Water activity depends on the structure of components as well as the surface activity of the product [30]. Sugars are known to hold onto water. The slight decrease in water activity was noted for gel with higher amount of AJC (Table 1). The significant difference in water content was observed for gels with a different addition of AJC but their water activity was similar. This means that only the high concentration of sugars may affect the degree of water binding by gels matrix. A similar trend was also observed for gels with chokeberry juice concentrate, the reduced water activity was related to the addition of a higher amount of concentrate [17].

The density of gels slightly increased with the addition of AJC and with the application of osmotic dehydration of gels in the solution with a higher amount of AJC (Table 1). The higher density of materials was related to an increase in dry matter in the gels, especially produced with the addition of concentrate at the amount 15–20%.

**Figure 1:**
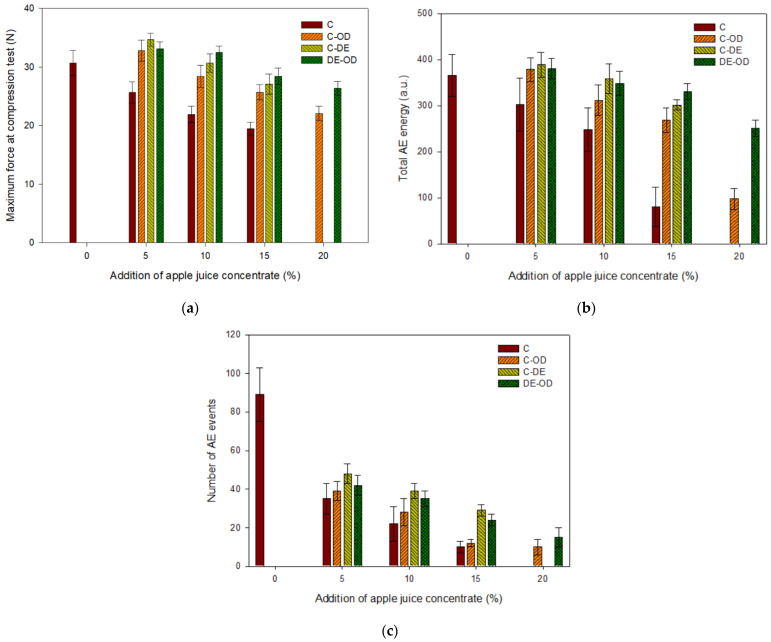
Effect of different composition and pre-treatment on the selected attributes of gels: (**a**) maximum force at compression test, (**b**) total AE energy, (**c**) number of AE events.

The mechanical tests showed that the maximum force (at compression test) gradually decreased with the addition of AJC in gel with or without the addition of maltodextrin (Figure 1a). A similar tendency was observed by Banerjee et al. [4]; the incorporation of carrot juice in the agar gel network caused a decrease in compression force and strain. Nussinovitch and Peleg [18] noted that an increase in strawberry pulp reduced the strength of gels. However, the addition of sugars to fruit based gels caused an increase in the material strength up to a certain level, but further addition of sugars decreased the hardness of samples. The presence of sugars increased the polymer interactions in gelled material but at high concentrations of sugars the non-homogenic, week and soft gels were produced. Sugars bound the free water in the gel samples. The presence of sucrose up to concentration of 40% led to stronger gels; however, the gel network collapsed at the sucrose level of 60% [31]. The addition of juice to agar-alginate products weakens the gel strength [32] which is in agreement with our results. The higher concentration of AJC and the addition of maltodextrin did not affect the failure strain. The effect of a higher concentration of sugars in the gels was observed after soaking of samples in the 20% solution of apple juice concentrate. In this case, the maximum force decreased and the failure strain increased in comparison with gel soaked in the 5% solution of AJC. This means that a less brittle gel with a weaker structure was formed. The high concentration of sugars can hinder the aggregation of hydrocolloid helices and it may reduce the size of junction zones [33] and the hardness of gels.

The effect of the osmotic dehydration of the hardness of gels with maltodextrin with the same amount of added AJC was not noticeable (Figure 1a). However, samples without maltodextrin soaked at the osmotic solution were considerably stronger than gels with added AJC (at the same concentration). Therefore, at high concentrations of sugars the reduction in the strength of gels can take place [3].

The acoustic emission descriptors such as amplitude of signal, duration of acoustic emission event, and the energy of AE event did not differ for investigated gels (data are not shown). However, the total acoustic emission energy and number of AE events decreased with the incorporation of a higher amount of concentrate (Figure 1b,1c). The changes in the total AE energy with an increase in AJC were lower for samples obtained with the addition of maltodextrin (Figure 1b). This may indicate that the presence of maltodextrin affected the stronger structure of gels. Some investigations have shown that the concentration of maltodextrin above 20% resulted in the formation of stronger gels [34,35]. The gelatin-maltodextrin composite gels exhibited a brittle failure behaviour which occurred at a lower strain than observed for the gelatin gels [36]. The presence of nuclei (aggregates of double helices of amylose) in maltodextrin gels created larger number of crystallites, which led to the rapid formation of a stronger gel. Increasing maltodextrin concentration from 15 to 40% caused a significant increase in the gel strength [37]. The maximum force for samples with the addition of maltodextrin was slightly higher than observed for gels without the carrier (at the same concentration of AJC in samples). It can be concluded that the gels with maltodextrin were more brittle and generated more acoustic events (more cracks) with a higher energy during the deformation of a stronger (harder) gel. The presence of maltodextrin in gels at the 15% addition of AJC affected the structure of gels and generation of a higher number of fractures then recorded for samples without the carrier (Figure 1c). The acoustic emission characteristics depends on the mechanical properties of the material and the presence of natural defects of raw material [38]. The effect of osmotic dehydration on acoustic properties of gels had a similar trend as was observed for maximum force. Gels prepared with the addition of AJC but without maltodextrin and the pre-treatment stage (soaking) emitted a lower acoustic energy which can be related to the less brittle structure of these soft gels.

**Figure 2:**
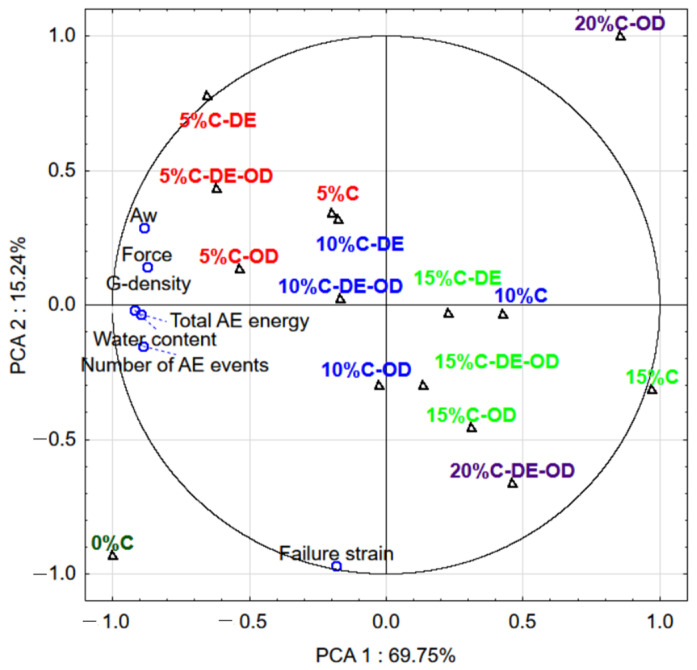
Biplot of first two components of PCA for properties of gel samples; 1 plot (○)—different attributes of gels, 2 plot (△)—samples with the different compositions of gels, the same colour of symbol indicates the same level of concentrations or additions of AJC.

The principal component analysis (PCA) showed that density was highly correlated with the acoustic parameters of samples (Figure 2). The maximum force can be a good indicator of the hardness of materials. Thus, the higher the hardness of gels, the more acoustic events and energy can be released during the deformation of sample. The density of gels correlated well with the water content and water activity of investigated samples. Gels with the same amount of added AJC and with maltodextrin or soaked in the solution with the same concentration of AJC presented similar properties, e.g., 10%C-OD and 10%C-DE-OD. Samples with a higher addition of AJC and agar gel (0%C) had different properties than other samples. This means that the incorporation of a high amount of AJC caused the creation of different a structure-texture product.

### 2.2. Characteristics of Dried Apple Gels

The freeze-drying method was used to dehydrate the gels obtained with different additions of AJC with or without maltodextrin. Pure agar gels and gels soaked at different concentrations of AJC were freeze-dried. The parameters and conditions of freeze drying were the same for all samples. The effect of different a composition of gel and applied pre-treatment on the selected physical and physio-chemical properties of the freeze-dried gel were analysed.

The water content of dried gel increased with the addition of AJC and after osmotic dehydration of the AJC solution with a higher concentration of sugars (Table 2). The lowest water content of dried gels was observed for samples with an addition of maltodextrin and after osmotic dehydration of gels (as the pre-treatment method before drying). Many investigations have shown the significant effect of the addition of biopolymers (mainly maltodextrins) on the sorption properties of sugar-rich foods such as dried fruit juices. The fruit freeze-dried products were characterised by very low values of glass transition temperature from 25 °C and −38 °C for water content at the range from 3.3 to 25%. Many dried fruit products obtained without carriers are in a rubbery state in room temperature conditions [39,40]. Table 2 shows that the water content of dried gels without carriers ranged from 4.46 to 17.42% which represents the same level of moisture content as obtained by other investigators for dried fruit products. Table 3 shows the results of differential scanning calorimetry (DSC) measurements of selected freeze-dried samples (with the lowest and the highest addition of AJC) in each group of samples with and without maltodextrin as well as soaked in AJC solutions. Data were collected as glass transitions determined on onset, midpoint, and endpoint. The glass transition in midpoint is frequently used as the T_g_. The highest T_g_ value was recorded for freeze-dried gels without other ingredients. The agar is a biopolymer of which the structure is based on disaccharides. Cooke et al. [41] obtained dried agar powder with the application of different drying methods and at low water activity (a_w_ = 0.33), T_g_ values varied between 85 and 105 °C. The glass transition obtained for freeze-dried gel was in a similar range (94.3 °C at a_w_ = 0.239) (Table 3). The increase in water content in dried gels caused the decrease in the glass transition temperature. The water content of freeze-dried gels with the addition of maltodextrin did not exceed 10% (Table 2). Incorporation of maltodextrin to the gel structure after drying led to an increase in T_g_ values (Table 3). The addition of maltodextrin as a biopolymer with high molecular weight affected the increase in glass transition temperature which limited the problems with sticking and caking [39,40].

Cassanelii et al. [42] observed the increase in water activity with an increase in solute content (up to 20% of sucrose). The freeze-dried gel with the higher concentrations of AJC is characterised by the higher values of water activity a_w_ = 0.4–0.6 (Table 2). This behaviour is related to the solute physical state (amorphous solid). Water in the freeze-dried materials with sugars performs the role of plasticizers. Water may enhance the time-dependent recrystallization process which leads to water desorption from samples and to the increase in water activity [42]. Application of soaking with the higher amount of AJC caused an increase in water content and water activity of freeze-dried gels. It may be concluded that the gels soaked in the lower amount of added concentrate contain the more crystalline forms of sugars which are not very hygroscopic. The water content of soaked gels and dried gels was lower than the samples prepared by direct addition of AJC to the agar solution. During osmotic dehydration, water diffuses from the gel to the solution with the increase in sugar concentration in the dehydrated material. The remaining free water was removed from the gels during the drying process. In case of dried gels with the addition of AJC, water was only removed during the freeze-drying process. The amount of sugars in the soaked and dried material was probably lower than in samples with added AJC. The dried samples with the addition of a lower amount of AJC (5%) with and without maltodextrin and produced with the application of soaking showed the low water content around 4–6% and glass transition temperature between 33.11 (5%C) and 61.63 °C (5%C-DE-OD). T_g_ temperatures obtained for these dried gels were higher than the storage temperature (25 °C). At low T_g_ (at the high water activity and the high concentration of AJC for dried gels) the presence of more hydrophilic groups caused higher hygroscopicity [43].

The water content of 17.42% and water activity of 0.601 obtained for 20%C-OD gel may not prevent the undesirable changes that occur at this level of moisture content. Between water activity of 0.4 to 0.7 changes in colour and texture as well as moisture migration can be observed. Almost all microbial activity is inhibited below water activity of 0.6 [44]. However, the value of a_w_ obtained for 20%C-OD gel was close to this limit. The Maillard reaction reached its peak at a_w_ = 0.6–0.7 [44]. This level of a_w_ did not ensure the stability of dried gel. Thus, 20%C-OD gel is not suitable for the practical application in production of dried snacks with fruit juice.

The apparent (geometric) and true density (pycnometric) increased with the incorporation of AJC by direct addition to the mixture or by osmotic dehydration of the gel in solution with a higher concentration of AJC (Table 2). However, the significant increase in geometric density was observed for dried gels with the highest addition or concentration of AJC due to a higher content of dry matter in applied solutions. The effect of the presence of sugars on the density of samples was noted for alginate gels immersed in sucrose solution for 48 h. Their apparent density after freeze drying (0.218–0.328 g∙cm^−3^) was higher than the dried samples prepared without infused sugars (0.193 g∙cm^−3^) [45].

The density of dried gels with the addition of a higher amount of AJC increased. The same trend was also observed for “wet gels”, the presence of sugar caused an increase in sample density.

The porosity of dried gels increased at a low addition AJC but a high amount of concentrate caused a decrease in porosity (Table 2). A similar effect was observed for freeze-dried gellan gel, the increase in sugar incorporation in gel led to a drop porosity from 84.8 to 48.6%. As the structure of freeze-dried gel (pore size distribution) depends on the freezing step, the difference between gels can be linked with a different supercooling mechanism. A higher degree of supercooling can be expected for sucrose which enhances the ice nucleation but limits the crystal growth [42]. Freeze-dried gels with a higher addition AJC were characterised by higher water content and water activity. Water can fill the pores present in the freeze-dried gel and reduce the size of pores as well as porosity of the material.

**Figure 3:**
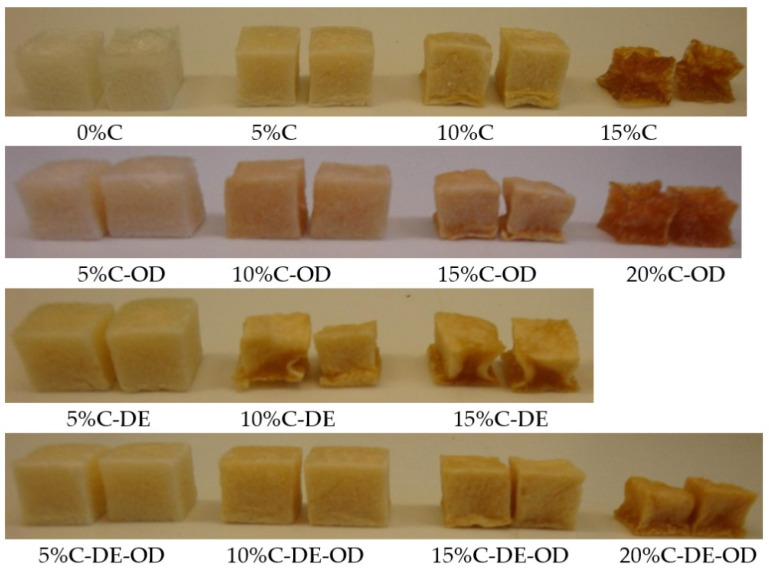
Images of investigated dried gels.

It can be noted that with a high presence of sugars, the structure of dried gels collapsed. The shrinkage represents the changes in volume of gel in comparison with dried samples. The shrinkage was larger with the application of a higher amount of AJC or with the use of a solution with a higher concentration of sugars. This high shrinkage of gels samples can be observed in Figure 3. The presence of low molecular substances in dried products during storage may result in the formation of the amorphous state which is the no-equilibrium state. Amorphous materials may change from a solid glassy to rubbery state while glass temperature is reached. Low molecular solids can show changes in physical properties and the structure of materials during storage. The significant changes in structure can be observed such as the collapse of structure with the increase in water content in the amorphous systems [46,47,48]. The stability of dried materials is related to water activity by the glass transition temperature (T_g_). Collapse of structure occurs at temperatures of 20 °C or more above the T_g_ [49]. The incorporation of high molecular weight biopolymers such as maltodextrin before drying may overcome this problem [50,51]. The moisture content of date syrup powders containing maltodextrin at different water activities were lower than those of date syrup. The addition of maltodextrin changed the balance of hydrophilic/hydrophobic sites which limited the amount of sorbed water. The increase in maltodextrin addition from 0 to 60% in date powders caused an increase in glass temperature from −1.27 to 31.11 °C [52,53]. The glass transition temperature of many fruit and vegetables powders as well as polymers decreased with increasing moisture content due to the plasticizing effect of water [54].

The reduced shrinkage and higher porosity of dried gels with addition of maltodextrin were observed for samples with a high concentration of AJC in comparison with samples without the incorporation of this carrier (Table 2).

**Figure 4:**
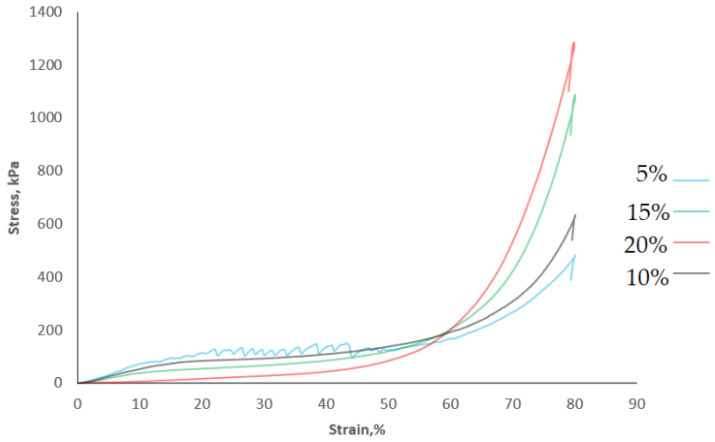
Compression curves of dried gels with addition of maltodextrin soaked in osmotic solution with 5, 10, 15, and 20% of AJC.

Figure 4 shows the example of the compression curves obtained for dried gels. The failure strain was equal to the maximum applied strain at compression for all samples (80%). The compression test showed that the dried samples with a higher addition of AJC were characterised by the lower values of forces at a smaller deformation (<30%), but the maximum compression stress at the final deformation was the highest for analysed samples. Nussinovitch et al. [45] also noted that the incorporation of sugars during the immersion of alginate gels caused an increase in stress of freeze-dried gels. The irregular jagged shape of stress-strain relationships obtained dried gels with an addition of a smaller amount of concentrate or for gels obtained by osmotic dehydration in 5% AJC (Figure 4) was typical for brittle cellular foods and crispy dehydrated gum gels. The concave shape of the compression curve was characteristic for samples obtained with the higher addition of AJC (Figure 4). The sigmoidal stress-strain curve was also observed for freeze-dried gels with orange juice and banana puree which was linked with the densification of products. Authors observed that the addition of fruit particles into the gels increased the bulk density and the thickness of solids’ edges and struts, but the porosity of dried gels decreased from 98 to 88% [8]. This indicates the plastic behaviour of dried gels with a high concentration of fruit juices [55]. A similar tendency was observed for the freeze-dried gels with the addition of apple juice concentrate.

**Figure 5:**
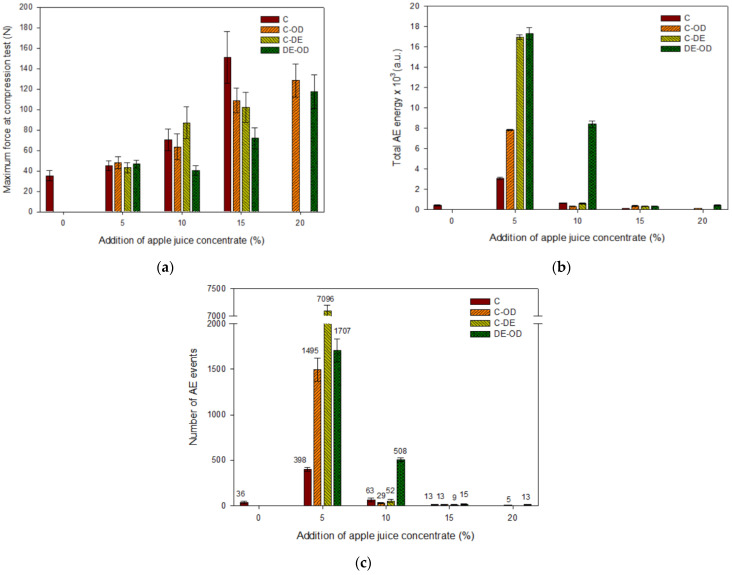
Effect of different composition and pre-treatment on the selected attributes of freeze-dried gels: (**a**) maximum force at compression test, (**b**) total AE energy, (**c**) number of AE events.

The mechanical properties of dried gels showed that the maximum force obtained at the compression test increased with an addition of AJC (Figure 5a). However, dried gel prepared with the addition of maltodextrin and soaked in AJC were less hard than other samples with 10 and 15% addition of concentrate. This dried gel also had lower shrinkage, water activity, and water content. The lower amount of infused sugars (form AJC) may affect the structure and thickness of pore walls.

The materials with a high T_g_ are harder and more brittle at an ambient temperature, but at a low T_g_ temperature the products are more elastic [56].

**Figure 6:**
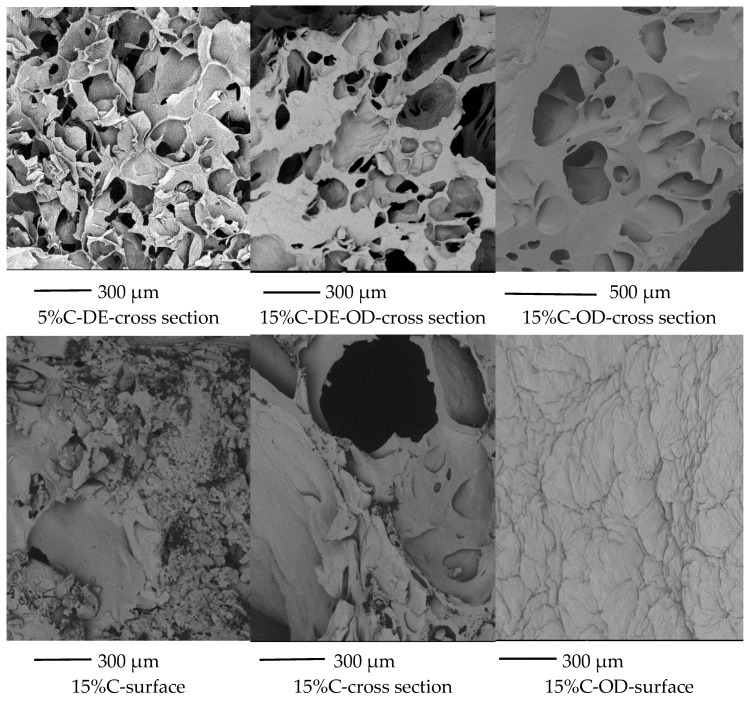
SEM microphotographs of dried gels.

The dried gels showed different mechanical properties than gel samples. The addition of a higher amount of concentrate caused a decrease in hardness with the decrease in the water content in gels. In the case of dried gels, the higher the amount of added AJC, the higher the compression force. However, samples with a lower content of water also showed lower values of maximum force. This effect was observed for dry cereal products and glassy polymers when the increase in water content and water activity caused the hardening of the samples (antiplasticizing effect of water). In some solid systems the increase in plasticizer addition leads to a harder structure despite T_g_ decrease. The situation may occur in amorphous cell foods. In some solid matrices small amounts of sorbed water led to an increase in the rigidity of samples [57,58]. Some researchers have stated that antiplastification can be observed due to “hole-filling” by the diluent [59]. SEM micrographs of dried agar gels with the higher addition of AJC showed the edges of pores were more round, and the thickness of walls increased in comparison with dried gels produced with a lower amount of AJC and without maltodextrin (Figure 6).

The acoustic descriptors of dried gels were characterised by high values of standard deviations which can be linked to the heterogenous structure of materials with many cracks, pores, and defects (Figure 5bc, Table 4). The acoustic emission energy of the event and the duration of the AE event did not differ for most dried gels samples. The amplitude of AE signal, total AE energy, and the number of AE events decreased with the incorporation of a higher amount of apple juice concentrate. The highest number of events with a high acoustic energy was observed for samples prepared with an addition of maltodextrin and the lowest amount of added AJC. The results of DSC showed that for these dried gels, the glass transition was considerably higher than the ambient temperature. It can be concluded that these dried gels behaved as brittle and crispy products. The incorporation of a higher amount of concentrate decreased the intensity of acoustic emission generated during the compression of dried samples. The effect of sugars addition on the reduction in acoustic activity during the deformation was observed in dried samples with a higher addition of AJC. Dried samples with a higher addition of AJC were harder but less brittle. The structure of dried gels collapsed and shrinkage was observed.

In the case of stored dried samples at different water activities, sugar crystallisation can occur. Amorphous sugars can absorb water vapour which leads to a decrease in T_g_ below the storage temperature, crystallisation starts, and the release of water can be observed. The process of sugar of crystallisation is time-dependent [60]. The properties of dried gels (glass transition and other mechanical properties and acoustic properties) were performed directly after drying. The texture-structure properties of dried gels were related to the composition of gels as well the changes in products during the freeze-drying process (the collapse of freeze-dried products with high concentrations of AJC and without maltodextrin). However, SEM microphotographs of surface and cross sections of dried gel with a 15% addition of AJC without maltodextrin showed the presence of coarse rough structures (Figure 6). This may indicate patrial sugar crystallisation. These structures (sugar crystals) were also observed for freeze-dried mango powders stored at the humidity of 55 and 66% [61]. The dried gels with 15% AJC addition showed high water activity a_w_ = 0.601 and compression force but very low values of glass transition.

The samples after freeze drying showed similar trend as observed for wet gels with the addition of AJC. The gels samples with 5% addition of AJC were characterised by the intensive acoustic energy with a high number of AE events. Gel samples prepared without soaking and addition of maltodextrin after drying generated a smaller number of events with lower energy. However, this effect was clearly evident for dried samples.

**Figure 7:**
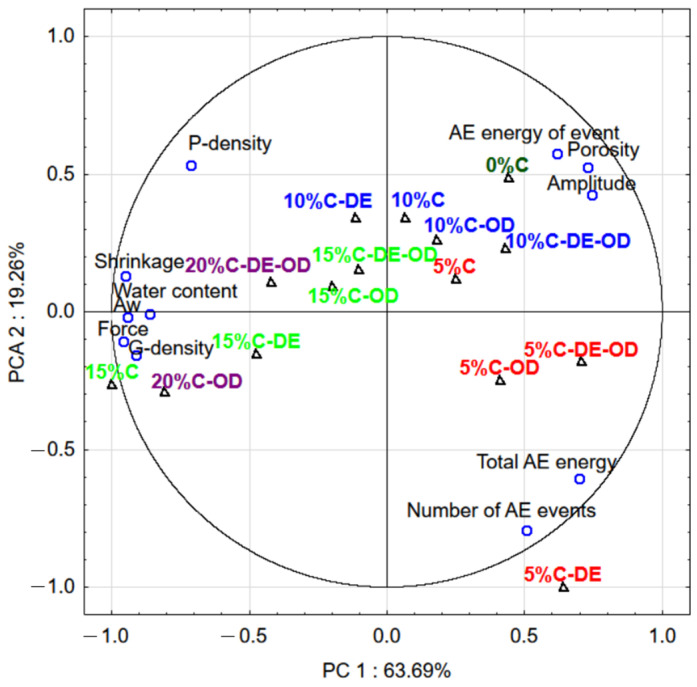
First two components of PCA for properties of dried gel samples1 plot (○)—different attributes of dried gels, 2 plot (△)—samples with the different compositions of dried gels, the same colour of symbol indicates the same level of concentrations or additions of AJC.

The principal component analysis (PCA) indicated the relation between investigated attributes (Figure 7). In the case of dried gels, the opposite trend was observed for the relation between the mechanical and acoustic properties than was noted for wet gels. Acoustic descriptors were negatively corelated with maximum force. The porosity of dried gels was positively correlated with the acoustic energy of events and their amplitude. The higher porosity and the presence of many pores in dried samples may generate more acoustic energy during the compression of samples. Force was positively correlated with water content and water activity which can be linked with the hardening of material with the presence of water. The increase in shrinkage caused the collapse of the gel matrix and the increase in the hardness of dried gel. The amount of added AJC affected the properties of dried gel to a greater extent than pre-treatment before the drying of gels. The high crispness is related to the higher number of acoustic events generated during the deformation of materials [22,27]. The addition of maltodextrin had a significant effect on the higher number of acoustic events (crispness) only for the dried gels with the lowest addition of AJC (5%C-DE).

## 3. Conclusions

The addition of apple juice concentrate changed the mechanical and acoustic properties of gels as well their water content. The higher concentration of AJC the softer gels were obtained. The pure agar gel was the most brittle sample. However, the addition of maltodextrin to agar gel enabled a stiffer product. Osmotic dehydration in apple juice concentrates also affected the higher hardness and stronger gel network in comparison with gel produced by the addition of AJC to the agar sol solution. The composition of gels as well the method or preparation of gel influenced the texture of dried gels and their glass transition temperature. The hardness of the freeze-dried gels increased with a higher amount of added AJC, samples with a lower concentration of sugars (the lower addition of AJC) were characterised by the lower shrinkage and higher porosity. This led to obtaining products with lower hardness and the higher crispness and more stability due to the high glass transition temperature. The addition of apple juice concentrate and maltodextrin may modify the texture-structure properties of dried gel. However, the amount of added concentrate should be low. The investigated mechanical and acoustic properties of dried gels showed the addition of apple concentrate at the level of 5% to agar solution was optimal. The incorporation of maltodextrin to the gel structure led to obtaining a less hard and more crispy product with a low water content.

## 4. Materials and Methods

### 4.1. Materials and Production of Gels and Dried Materials

The gels were produced through the following ingredients: agar-agar powder (Hortimex Sp. z o.o., Konin, Poland), apple juice concentrate (70.5 Brix) (Binder International, Tarczyn, Poland), and maltodextrin DE 10–13 (Pepees S.A., Łomża, Poland). The gels were prepared using two different methods. Table 5 presents the composition of gels obtained with the addition of apple juice concentrate to sol solution (dissolved gelling agent in water). The agar powder with or without maltodextrin was dispersed in distilled water, heated to 90 °C and continuously stirred at a speed of 80 rpm. The solution was cooled in the water bath until 60 °C and the required amount of apple juice concentrate was added and stirred. The mixture was poured into Petri dishes and allowed to set at temperature of 4 °C. After 24 h of storage, the gels were diced in to 10 mm cubes. A constant ratio of 1:0.5 was kept between juice solids and maltodextrin. Additionally, one type of gel (with composition presented in Table 6) was obtained by soaking gel cubes in the solution of diluted apple juice concentrate with concentration of 5, 10, 15, and 20%. The mass ratio of gels to the apple concentrate solution was kept at 1:4. After 24 h of soaking, the gels were blotted dry.

In the next step, the gels were frozen at −40 °C for 1 h using a shock freezer (Irinox, Corbanese, Italy). Samples were freeze-dried under the pressure of 63 Pa and at a shelf temperature of 20 °C with an application of Gamma 1–16LSC freeze-dryer (Martin Christ Gefriertrocknungsanlagen GmbH, Osterode am Harz, Germany).

### 4.2. Measurements of Properties of Gels and Dried Materials

Water activity was measured using a Hygrolab C laboratory analyser (Rotronic, Bassersdorf, Switzerland) with a measurement accuracy of ±0.001. The measurement of the moisture content of gels after drying was carried out with the application of the vacuum drying method at the temperature of 80 °C and pressure of 1 kPa for 24 h. The measurements were repeated in triplicate.

The geometric (apparent) density of the gels was calculated based on the volume and mass of samples. The dimension of samples (cubes) was measured with a calliper, and the volume of the cubes were calculated (*V_o_*). The volume of dried gels (*V_f_*) was measured using the toluene displacement method [62]. The pycnometric (true) density was measured using a helium stereopycnometer (Quantachrome Instruments, Boynton Beach, FL, USA). The porosity of dried gels was calculated according to the formula:

$$\varepsilon =\left(1-\frac{\rho_{g}}{\rho_{p}}\right)\cdot 100\%$$

where: *ε* is the porosity, %; *ρ_p_* is the pycnometric density of dried gels, g·cm^−3^; *ρ_g_* is the geometric density of dries gels, g·cm^−3^.

Shrinkage of samples *S_v_* was calculated as:

$$S_{v}=\left(1-\frac{V_{f}}{V_{o}}\right)$$

where: *V_o_* is the volume of gel, cm^−3^; *V_f_* is the volume of dried gel, cm^−3^.

The mechanical properties of gels were measured using a TA-HD plus texture analyser (5-kg load cell) and flat-type probe (Stable Micro Systems, Surrey, UK). Samples (cubes with a side of 10 mm) were compressed at a constant speed of 1 mm∙s^−1^ and up to strain 80%. The maximum force (N) and the failure strain (%) was recorded for gels. Compression curves present the relationship between stress and strain. Strain was calculated as the ratio of absolute deformation (mm) and the specimen’s initial height (mm). Stress (kPa) was defined as the ratio of force and the cross-sectional area of samples. Twenty individual samples were subjected to a compression test.

The measurement of the acoustic emission (AE) was carried out during the compression of the gels and dried samples. Acoustic emission signal from the range 0.1–16 kHz was recorded using a piezoelectric accelerometer type 4381 (Brüel and Kjær Naerum, Denmark). The total number of AE events and total AE energy (arbitrary unit- a.u.) were recorded for gels and analysed according to the protocol described by Jakubczyk et al. [63]. Additionally, the analysis of acoustic emission signal enabled to obtain AE energy of the event (pJ) and the signal amplitude (mV) [22]. Acoustic measurements were carried out in 20 replications.

The glass transition temperature of the selected dried gels was measured using the Q200 DSC (TA Instruments, New Castle, DE, USA) according to protocol described by Ostrowska-Ligęza et al. [64]. Samples were heated from −50 to 150 °C at a heating rate of 3 °C/min. The onset, midpoint, and endpoint glass transition temperature were determined.

The SEM structure of selected dried samples were analysed with the application of Quanta 200 ESM (FEI Company, Hillsboro, OR, USA) according to procedure applied by Jakubczyk et al. [9].

One-way analysis of variance (ANOVA) and paired Tukey’s Honest Significant Difference method test were used to establish the significance of differences among samples at the 95% significance level. Principal component analysis (PCA) was applied to describe relations between the different parameters of analysed samples of gels as well as dried materials. The results are presented as a biplot. The STATISTICA software v. 12.5 (StatSoft Inc., Tulsa, OK, USA) was applied to statistical analysis of data.

## Figures and Tables

**Table 1:** The selected physical properties of gels.

Samples	Water Content, %	Water Activity	Geometric Density, g∙cm^−3^	Failure Strain, %
0%C	98.1 ± 0.1 ^a^*	0.991 ± 0.004 ^ab^	1.12 ± 0.03 ^g^	40.1 ± 1.2 ^a^
5%C	93.6 ± 0.2 ^c^	0.988 ± 0.001 ^bc^	1.14 ± 0.01 ^fg^	35.4 ± 0.9 ^ef^
10%C	87.4 ± 0.1 ^g^	0.985 ± 0.002 ^bc^	1.20 ± 0.01 ^cdef^	36.4 ± 0.8 ^cdef^
15%C	83.4 ± 0.2 ^l^	0.983 ± 0.001 ^c^	1.23 ± 0.02 ^abcd^	37.4 ± 0.5 ^bcde^
5%C-OD	95.4 ± 0.4 ^b^	0.988 ± 0.003 ^bc^	1.13 ± 0.01 ^g^	36.4 ± 1.4 ^cdef^
10%C-OD	90.6 ± 0.1 ^e^	0.986 ± 0.001 ^bc^	1.18 ± 0.03 ^defg^	37.8 ± 0.5 ^bcd^
15%C-OD	88.1 ± 0.1 ^g^	0.985 ± 0.003 ^bc^	1.17 ± 0.02 ^defg^	38.4 ± 0.4 ^abc^
20%C-OD	85.3 ± 0.2 ^h^	0.983 ± 0.002 ^c^	1.20 ± 0.01 ^cdef^	32.4± 0.1 ^g^
5%C-DE	92.1 ± 0.2 ^d^	0.994 ± 0.003 ^a^	1.16 ± 0.02 ^efg^	35.0 ± 0.8 ^f^
10%C-DE	89.0 ± 0.5 ^f^	0.990 ± 0.003 ^abc^	1.23 ± 0.03 ^abcd^	35.9 ± 0.6 ^def^
15%C-DE	85.9 ± 0.4 ^h^	0.987 ± 0.002 ^bc^	1.27 ± 0.01 ^ab^	36.9 ± 0.4 ^cdef^
5%C-DE-OD	93.8 ± 0.2 ^c^	0.993 ± 0.002 ^ab^	1.15 ± 0.02 ^efg^	36.0 ± 0.3 ^def^
10%C-DE-OD	89.1 ± 0.1 ^f^	0.988 ± 0.005 ^abc^	1.21 ± 0.03 ^bcde^	37.0 ± 0.5 ^bcdef^
15%C-DE-OD	87.4 ± 0.2 ^g^	0.986± 0.002 ^bc^	1.25 ± 0.01 ^abc^	37.9 ± 0.3 ^bcd^
20%C-DE-OD	85.3 ± 0.4 ^h^	0.984 ± 0.002 ^c^	1.29 ± 0.02 ^a^	39.1 ± 0.7 ^ab^

* The different letters in the columns indicate the significant difference between the obtained values for samples *p* ≤ 0.05.

**Table 2:** Selected physical and physio-chemical parameters of dried gels.

Samples	Water Content, %	Water Activity	Geometric Density, g∙cm^−3^	Pycnometric Density, g∙cm^−3^	Porosity, %	Shrinkage
0%C	8.12 ± 0.22 ^d^*	0.239 ± 0.003 ^g^	0.16 ± 0.04 ^i^	0.65 ± 0.02 ^h^	77.4 ± 3.6 ^a^	0.258 ± 0.032 ^ef^
5%C	5.69 ± 0.03 ^g^	0.245 ± 0.002 ^fg^	0.44 ± 0.01 ^fgh^	1.12 ± 0.04 ^d^	61.2 ± 0.5 ^cd^	0.152 ± 0.061 ^gh^
10%C	6.24 ± 0.15 ^g^	0.249 ± 0.009 ^fg^	0.47 ± 0.01 ^fg^	1.27 ± 0.05 ^c^	63.3 ± 0.5 ^c^	0.432 ± 0.021 ^cd^
15%C	17.42 ± 0.62 ^a^	0.601 ± 0.002 ^a^	0.95 ± 0.01 ^a^	1.28 ± 0.03 ^c^	26.0 ± 0.5 ^h^	0.753 ± 0.042 ^a^
5%C-OD	4.46 ± 0.09 ^h^	0.158 ± 0.006 ^i^	0.51 ± 0.05 ^ef^	0.99 ± 0.01 ^e^	50.2 ± 2.9 ^ef^	0.065 ± 0.015 ^hi^
10%C-OD	6.96 ± 0.12 ^f^	0.277 ± 0.001 ^e^	0.41 ± 0.03 ^gh^	1.17 ± 0.01 ^d^	65.8 ± 1.5 ^bc^	0.199 ± 0.034 ^fg^
15%C-OD	7.76 ± 0.46 ^e^	0.306 ± 0.003 ^d^	0.57 ± 0.01 ^de^	1.34 ± 0.01 ^c^	57.7 ± 0.4 ^cd^	0.414 ± 0.042 ^cd^
20%C-OD	12.17 ± 0.03 ^b^	0.451 ± 0.002 ^b^	0.99 ± 0.01 ^a^	1.42 ± 0.01 ^ab^	30.5 ± 0.4 ^h^	0.601 ± 0.022 ^b^
5%C-DE	4.32 ± 0.13 ^h^	0.184 ± 0.013 ^h^	0.42 ± 0.03 ^gh^	0.75 ± 0.02 ^g^	45.3 ± 2.3 ^fg^	0.012 ± 0.017 ^i^
10%C-DE	8.36 ± 0.16 ^de^	0.400 ± 0.008 ^c^	0.64 ± 0.01 ^cd^	1.31 ± 0.01 ^c^	51.4 ±0.4 ^e^	0.595 ± 0.021 ^b^
15%C-DE	8.13 ± 0.14 ^e^	0.401 ± 0.003 ^c^	0.74 ± 0.01 ^b^	1.30 ± 0.03 ^c^	43.3 ± 0.4 ^g^	0.516 ± 0.043 ^bc^
5%C-DE-OD	4.30 ± 0.21 ^h^	0.137 ± 0.002 ^j^	0.36 ± 0.04 ^h^	0.89 ± 0.01 ^f^	61.0 ± 2.6 ^cd^	0.072 ± 0.053 ^hi^
10%C-DE-OD	3.96 ± 0.14 ^h^	0.149 ± 0.005 ^ij^	0.44 ± 0.04 ^fgh^	1.40 ± 0.04 ^ab^	69.5 ±1.6 ^b^	0.099 ± 0.032 ^ghi^
15%C-DE-OD	5.99 ± 0.05 ^g^	0.258 ± 0.009 ^f^	0.71 ± 0.03 ^bc^	1.41 ± 0.04 ^ab^	50.4 ±1.2 ^ef^	0.357 ± 0.019 ^de^
20%C-DE-OD	9.78 ± 0.07 ^c^	0.403 ± 0.007 ^c^	0.79 ± 0.01 ^b^	1.43 ± 0.02 ^a^	45.0 ± 0.4 ^g^	0.507 ± 0.026 ^bc^

* The different letters in the columns indicate the significant difference between the obtained values for samples, *p* ≤ 0.05.

**Table 3:** Glass transition temperature (T_g onset_, T_g midpoint,_ T_g endpoint_) of selected freeze-dried gels.

Samples	T_g onset_	T_g midpoint_	T_g endpoint_
0%C	88.74	94.03	98.12
5%C	27.90	33.11	39.41
15%C	−35.24	−24.36	−29.75
5%C-OD	46.13	52.75	59.00
15%C-OD	9.99	16.91	23.82
5%C-DE	40.43	44.68	48.93
15%C-DE	−2.93	0.54	8.01
5%C-DE-OD	56.15	61.63	67.11
15%C-DE-OD	7.00	12.56	18.13
20%C-DE-OD	−24.44	−16.32	−20.81

**Table 4:** Selected acoustic parameters of dried gels.

Samples	AE Energy of Event, pJ	Amplitude, mV
0%C	3890.3 ± 1779.0 ^a^*	545.7 ± 162.9 ^ab^
5%C	2358.5 ± 832.6 ^ab^	457.0 ± 160.3 ^ab^
10%C	4186.3 ± 1655.6 ^a^	718.7 ± 181.9 ^a^
15%C	1232.2 ± 745.2 ^ab^	125.3 ± 32.1 ^cde^
5%C-OD	1767.4 ± 641.9 ^ab^	425.1 ± 85.6 ^abc^
10%C-OD	3407.3 ± 1608.7 ^ab^	497.50 ± 52.8 ^ab^
15%C-OD	1699.5 ± 922.1 ^ab^	353.5 ± 51.5 ^bcde^
20%C-OD	452.1 ± 99.4 ^b^	85.4 ± 11.1 ^e^
5%C-DE	2013.2 ± 935.4 ^ab^	458.0 ± 118.0 ^ab^
10%C-DE	3688.2 ± 999.9 ^a^	681.7 ± 147.6 ^a^
15%C-DE	1201.1 ± 101.1 ^ab^	98.8 ± 14.7 ^ed^
5%C-DE-OD	3887.7 ± 840.4 ^a^	675.6 ± 88.9 ^ab^
10%C-DE-OD	2872.9 ± 804.8 ^ab^	603.7 ± 159.3 ^ab^
15%C-DE-OD	2349.8 ± 936.5 ^ab^	403.9 ± 54.3 ^abcde^
20%C-DE-OD	2321.1 ± 935.6 ^ab^	416.0 ± 64.8 ^abcd^

* The different letters in the columns indicate the significant difference between the obtained values for samples, *p* ≤ 0.05.

**Table 5:** Composition of gels with addition of apple juice concentrate.

Type of Gel	Apple Juice Concentrate, %	Agar Powder, %	Maltodextrin, %	Water, %
0%C	0	2.0	0.0	98.0
5%C	5	2.0	0.0	93.0
10%C	10	2.0	0.0	88.0
15%C	15	2.0	0.0	83.0
5%C-DE	5.0	2.0	1.8	91.2
10%C-DE	10.0	2.0	3.5	84.5
15%C-DE	15.0	2.0	5.0	78.0

**Table 6:** Composition of gels before soaking in 5, 10, 15, and 20% solution of apple juice concentrate.

Type of Gel	Agar Powder, %	Maltodextrin, %	Water, %
5%C-OD	2.0	0.0	98.0
10%C-OD	2.0	0.0	98.0
15%C-OD	2.0	0.0	98.0
20%C-OD	2.0	0.0	98.0
5%C-DE-OD	2.0	1.8	96.2
10%C-DE-OD	2.0	3.5	94.5
15%C-DE-OD	2.0	5.0	93.0
20%C-DE-OD	2.0	7.0	91.0

## Data Availability

The data generated or analysed during this study are available from the corresponding author on reasonable request.
